# Activation of Muscarinic M1 Acetylcholine Receptors Induces Long-Term Potentiation in the Hippocampus

**DOI:** 10.1093/cercor/bhv227

**Published:** 2015-10-15

**Authors:** Siobhan H. Dennis, Francesca Pasqui, Ellen M. Colvin, Helen Sanger, Adrian J. Mogg, Christian C. Felder, Lisa M. Broad, Steve M. Fitzjohn, John T.R. Isaac, Jack R. Mellor

**Affiliations:** 1 Neuroscience, Eli Lilly & Company, Windlesham, Surrey GU20 6PH, UK; 2 Neuroscience, Eli Lilly & Company, Indianapolis, IN 46285, USA; 3 School of Physiology and Pharmacology, University of Bristol, Bristol BS8 1TD, UK

**Keywords:** CA1, hippocampus, long-term potentiation, muscarinic m1 receptor, synaptic plasticity

## Abstract

Muscarinic M1 acetylcholine receptors (M1Rs) are highly expressed in the hippocampus, and their inhibition or ablation disrupts the encoding of spatial memory. It has been hypothesized that the principal mechanism by which M1Rs influence spatial memory is by the regulation of hippocampal synaptic plasticity. Here, we use a combination of recently developed, well characterized, selective M1R agonists and M1R knock-out mice to define the roles of M1Rs in the regulation of hippocampal neuronal and synaptic function. We confirm that M1R activation increases input resistance and depolarizes hippocampal CA1 pyramidal neurons and show that this profoundly increases excitatory postsynaptic potential-spike coupling. Consistent with a critical role for M1Rs in synaptic plasticity, we now show that M1R activation produces a robust potentiation of glutamatergic synaptic transmission onto CA1 pyramidal neurons that has all the hallmarks of long-term potentiation (LTP): The potentiation requires NMDA receptor activity and bi-directionally occludes with synaptically induced LTP. Thus, we describe synergistic mechanisms by which acetylcholine acting through M1Rs excites CA1 pyramidal neurons and induces LTP, to profoundly increase activation of CA1 pyramidal neurons. These features are predicted to make a major contribution to the pro-cognitive effects of cholinergic transmission in rodents and humans.

## Introduction

Cholinergic projections from the medial septum play an important role in the function of the hippocampus. The release of acetylcholine in the hippocampus during exploration as well as REM sleep activates muscarinic and nicotinic receptors that regulate the processing of information by hippocampal circuits (Hasselmo 2006; Teles-Grilo Ruivo and Mellor 2013). Hippocampus-dependent memory is disrupted by pharmacological inhibition or genetic ablation of muscarinic receptors (Blokland et al. 1992; Anagnostaras et al. 2003; Atri et al. 2004; Wess 2004; Green et al. 2005) and, conversely, enhancing endogenous acetylcholine with acetylcholinesterase inhibitors in Alzheimer's disease (McGleenon et al. 1999) or activation of M1 muscarinic receptors (M1Rs) in cognitively impaired humans (Nathan et al. 2013) can improve memory. Because of the hypothesized importance of synaptic plasticity to memory processes, it is proposed that acetylcholine release enhances learning by modulating the induction and expression of synaptic plasticity (Hasselmo 2006). Indeed, the induction of hippocampal synaptic plasticity during learning requires muscarinic receptor activation (Mitsushima et al. 2013).

M1Rs are proposed to mediate many of the actions of acetylcholine in the hippocampus, where they are expressed predominantly in excitatory neurons on both somatic and dendritic compartments (Levey et al. 1995; Yamasaki et al. 2010), as well as some expression in inhibitory interneurons (Cea-del Rio et al. 2010; Yi et al. 2014). However, the precise roles of M1Rs have been difficult to define due to the lack, until recently, of ligands with sufficient selectivity to unambiguously identify the roles of M1Rs in brain slices or in vivo. The recently developed ligands, in combination with the availability of M1R knock-out (M1R KO) mice, now enable such studies to be performed. Indeed, recent work shows that M1Rs play an important role in hippocampus-dependent learning and memory through their ability to strongly depolarize hippocampal pyramidal neurons and by facilitating the induction of long-term synaptic plasticity (Anagnostaras et al. 2003; Wess 2004; Shinoe et al. 2005; Dickinson et al. 2009; Bradley et al. 2010; Buchanan et al. 2010; Dasari and Gulledge 2011; Digby et al. 2012). M1Rs depolarize hippocampal pyramidal neurons by inhibiting voltage-dependent Kv7 potassium channels that mediate the “M-current” (Dasari and Gulledge 2011) and likely promote back propagation of action potentials into the dendrites to promote long-term potentiation (LTP) induction (Tsubokawa and Ross 1997; Petrovic et al. 2012). However, M1Rs have also recently been shown to enhance NMDA receptor (NMDAR) activity in CA1 pyramidal neurons by inhibiting SK potassium channels located on postsynaptic spines that negatively regulate NMDAR function (Buchanan et al. 2010; Giessel and Sabatini 2010). This second mechanism also plays an important role in promoting NMDAR-dependent synaptic plasticity.

Here, we have further investigated the roles of M1Rs in hippocampus using recently developed M1R-selective agonists: 77-LH-28-1 and GSK-5 (Langmead et al. 2008; Budzik et al. 2010). We use them in combination with M1R KO mice to show that application of M1R agonist to adult hippocampal slices produces a robust potentiation of glutamatergic synaptic transmission on CA1 pyramidal neurons that is NMDAR-dependent and bi-directionally occludes with synaptically induced LTP.

## Materials and Methods

### Ethical Approval

All animal procedures and experiments were conducted in accordance with the United Kingdom Animals (Scientific Procedures) Act 1986 and EU Directive 2010/63/EU 2010. All experimental protocols were approved by the British National Committee for Ethics in Animal Research.

### Native Mouse Membrane Preparation

All procedures were performed at 4°C. Tissue samples were homogenized in sucrose buffer (10 mm HEPES, 1 mm EGTA, 1 mm DTT, 10% sucrose and 1 tablet/50 mL Complete Protease Inhibitor Cocktail; pH 7.4) using an electric IKA RW20 (800 rpm) with glass/teflon homogenizer. Homogenate was centrifuged at 1000×*g* for 10 min and supernatant collected, the pellet was rehomogenized and centrifuged again, as above, and supernatant pooled and centrifuged at 11 000×*g* for 20 min. The resulting pellet was suspended in a final storage buffer (10 mm HEPES, 1 mm EGTA, 1 mm MgCl_2_, 1 mm DTT; pH 7.4) and centrifuged at 27 000×*g* for 20 min. Supernatant was removed and the final pellet suspended in 2 mL of final storage buffer. Protein concentration was measured using the Bradford method (Bradford 1976) (Coomassie Plus, Bio-Rad protein assay kit) with bovine gamma globulin standards. Samples were then aliquoted and stored at −80°C.

### Native Mouse GTPɣ[^35^S] Binding Assays

GTPɣ[^35^S] binding in mouse WT and M1 KO hippocampal membranes were determined in triplicate using an antibody capture technique in 96-well plate format (DeLapp et al. 1999). Membrane aliquots (15 µg/well) from WT or M1 KO C57BL6/NTac mice were incubated with test compound and GTPɣ[^35^S] (500 pM/well) for 30 min. Labeled membranes were then solubilized with 0.27% Nonidet P-40 plus Gqα antibody (E17, Santa Cruz) at a final dilution of 1:200 and 1.25 mg/well of anti-rabbit scintillation proximity beads. Plates were left to incubate for 3 h and then centrifuged for 10 min at 2000 rpm. Plates were counted for 1 min/well using a Wallac MicroBeta Trilux scintillation counter (PerkinElmer). All incubations took place at room temperature in GTP-binding assay buffer (In mm, 20 HEPES, 100 NaCl, 5 MgCl_2_; pH 7.4).

### FLIPR-Based Human and Rat mAChR Assays

CHO cells stably expressing recombinant human M1, M3, and M5 Rs and AV12 cells stably expressing Gα15 and recombinant human M2 or M4 Rs were cultured in DMEM with high glucose and pyridoxine hydrochloride supplemented with 5–10% heat-inactivated fetal bovine serum, 10–20 mm HEPES, 1 mm l-glutamine, 1% penicillin/streptomycin solution and selection agents, 0.5 mg/mL geneticin, or 0.3 µg/mL puromycin. Confluent cultures were passaged weekly and cells harvested 24 h prior to assay using 0.25% trypsin–EDTA and plated at a density of 40 000–50 000 cells per well in tissue culture treated, poly-d-lysine-coated 96-well black-walled, clear bottom plates (Corning or Becton-Dickinson). For FLIPR (FLIPR-tetra, Molecular Devices) assays, media was removed and cells were incubated with 5 µm Fluo-4-AM/0.05% pluronic F-127 (Invitrogen) in a HEPES-buffered salt solution (HEPES-HBSS; composition, in mm; 135 NaCl, 5 KCl, 1.3 CaCl_2_, 0.5 MgCl_2_, 0.4 MgSO_4_, 0.4 KH_2_PO_4_, 4.2 NaHCO_3_, 0.3 Na_2_HPO_4_, 5.6 glucose, 20 HEPES, +2.5 mm probenecid for CHO cell lines, pH 7.5 adjusted with 5 m NaOH) for 1 h at room temperature, in the dark, before the media was removed and replaced with HEPES-buffered salt solution in the absence of Fluo-4. Probenecid was included to optimize dye loading in CHO cell lines. Although probenecid has been reported to interact and activate some TRP channels {McClenaghan, 2012 #2996}, there are no reports of interactions with mAChRs or nontransfected CHO cells. Plates were then transferred to FLIPR for experiments, which were also conducted at room temperature.

For data analysis, relative EC_50_ and IC_50_ values were calculated using a 4-parameter logistic curve (GraphPad Prism v6).

### Electrophysiology

#### Slice Preparation

Transverse hippocampal slices were prepared from adult (6–9 weeks old) male C57/BL6J, wild-type (WT), or M1R KO mice (Fisahn et al. 2002) (Line 1784, Taconic), or Lister hooded rats (Charles River). Slices from mice were used for the data shown in Figures 2, 4, 5 and 6 and slices from rats used in Figure 3. Brains were immediately removed following cervical dislocation (mice) or isoflurane anesthetization and decapitation (rats) and immersed in ice-cold cutting artificial cerebral spinal fluid (aCSF) containing (in mm): 119 NaCl, 10 glucose, 26 NaHCO_3_, 2.5 KCl, 1 NaH_2_PO_4_, 0.65 CaCl_2_, and 3 MgSO_4_ (mice) or 87 NaCl, 75 sucrose, 25 d-glucose, 25 NaHCO_3_, 2.5 KCl, 1.25 NaH_2_PO_4_, 0.5 CaCl_2_, and 7 MgCl_2_ (rats). Individual hippocampi were mounted on agar and 350-μm (mice) or 400-µm (rats)-thick slices were cut using a microslicer (VT1200S, Leica Microsystems). Following preparation, slices were transferred to aCSF containing (in mm): 119 NaCl, 10 d-glucose, 26 NaHCO_3_, 2.5 KCl, 1 NaH_2_PO_4_, 1.3 MgSO_4_, and 2.5 CaCl_2_ (mice) or 124 NaCl, 3 KCl, 26 NaHCO_3_, 1.25 NaH_2_PO_4_, 3 ascorbic acid, 1 MgSO_4_, 10 d-glucose and 2 CaCl_2_ (rats), maintained at 35°C for 30 min and then stored at room temperature for a further 30 min before recording. All solutions were saturated with 95% O_2_ and 5% CO_2_ and had osmolarity of 300–310 mOsm.

#### Recording

Slices were placed in a submerged recording chamber perfused with aCSF at 33°C at 4–6 mL/min. CA1 pyramidal cells were visualized using IR-DIC optics. Patch electrodes with a resistance of 4–5 MΩ were pulled from borosilicate filamented glass capillaries and filled with intracellular solution containing (in mm) 120 KMeSO_3_, 10 HEPES, 0.2 EGTA, 4 Mg-ATP, 0.3 Na-GTP, 8 NaCl, and 10 KCl, pH 7.4, 280–285 mOsm. Extracellular recording electrodes were filled with aCSF (resistance 1–3 MΩ). Bridge balance was employed for all whole-cell current clamp recordings, and access and input resistances were monitored throughout experiments from 500 ms, 20 pA current injections. Excitatory postsynaptic potentials (EPSPs) were recorded in the presence of picrotoxin (50 µm) and CGP55845 hydrochloride (1 µm) to block GABA_A_ and GABA_B_ receptors, respectively, with cells maintained in current clamp at −70 mV. Inhibitory postsynaptic potentials (IPSPs) were recorded in the presence of NBQX (3 µm) and L, 689–560 (5 µm) to block AMPA and NMDARs, respectively, with cells held at −55 mV. No junction potential correction was applied (calculated at −9.1 mV). Intracellular recordings were digitized at 80 kHz and filtered at 20 kHz. Extracellular recordings were digitized at 10 kHz and filtered at 3 kHz. All recordings were made using Molecular Devices 700B amplifiers. Synaptic responses were evoked using bipolar stimulating electrodes placed in stratum radiatum. For two pathway experiments, stimulating electrodes were placed either side of the recording electrode. Theta burst stimulation to induce LTP consisted of 10 bursts at 5 Hz where each burst consisted of 5 stimuli at 100 Hz. All experiments within groups were interleaved and performed with experimenter blind to the animal genotype.

#### Data Analysis

All data are expressed as mean ± s.e.m. Example traces shown are averages of 10–20 consecutive sweeps. Statistical significance was assessed using paired or unpaired *t-*tests as appropriate and the level of significance set at *P* < 0.05. Synaptic strength for extracellular recordings was measured as the initial slope of the field potential response. The independence of synaptic pathways was determined after LTP experiments by combinatorial paired-pulse experiments (interstimulus interval 40 ms) and data omitted from analysis if significant cross-pathway facilitation was observed.

### Drugs

L-689,560, Picrotoxin, NBQX, D-AP5, and CGP55845 hydrochloride were purchased from Tocris, UK. Compounds were dissolved in DMSO, except picrotoxin and D-AP5, which were dissolved in water. Compounds were separated into appropriate aliquots and stored at −20°C.

GTPɣ[^35^S] and Anti-rabbit SPA beads were purchased from PerkinElmer, UK, Gqα antibody (E17) was from Santa Cruz Biotechnology, Nonidet P-40 10% solution from Roche Applied Sciences, Dithiothreitol (DTT) from Sigma, UK, and Complete protease inhibitor cocktail purchased from Roche Applied Sciences

GSK-5 (Compound 5 from [Budzik et al. 2010]) and 77-LH-28-1 (1-[3-(4-butyl-1-piperidinyl)propyl]-3,4-dihydro-2[1H]-quinolinone) were synthesized in-house at Eli Lilly and Company Ltd.

## Results

### M1R Selectivity of Allosteric Agonists 77-LH-28-1 and GSK-5

**Figure 1.**
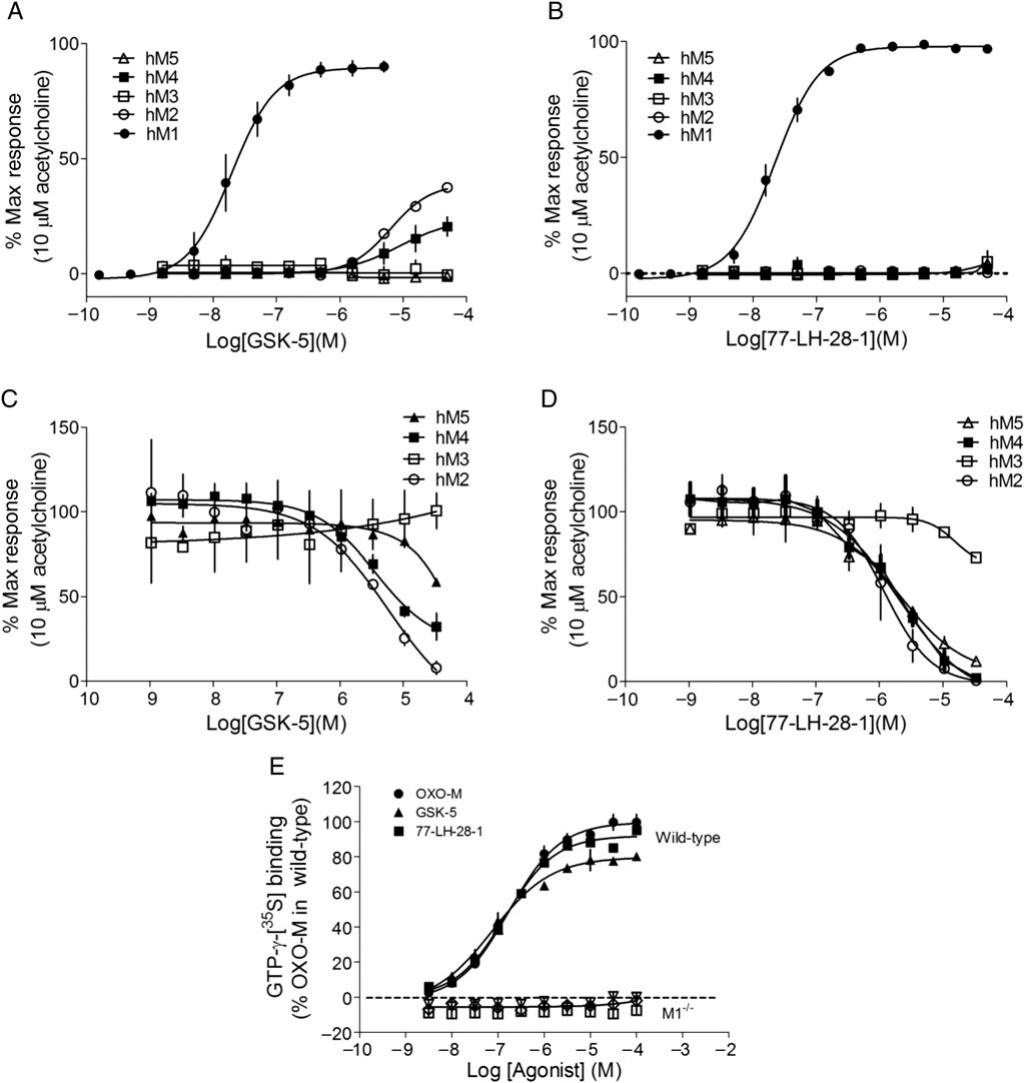
Selectivity of M1R agonists 77-LH-28-1 and GSK-5. (*A–D*) Intracellular calcium responses in CHO cells expressing recombinant human M1–M5 muscarinic receptors in response to application of GSK-5 (*A*) or 77-LH-28-1 (*B*) compared with a maximal response to 10 µm acetylcholine. Intracellular Ca^2+^ responses to 10 µm acetylcholine in the presence of GSK-5 (*C*) or 77-LH-28-1 (*D*) illustrate antagonism at muscarinic receptors. Data are plotted as mean ± s.e.m, *n* = 3 independent experiments. (*E*) GTPɣ [^35^S] binding to Gα_q_ in membranes prepared from WT (closed symbols) or M1R KO (open symbols) mouse hippocampi was measured after application of oxotremorine M (oxo-m), 77-LH-28-1 or GSK-5. Data are shown as a percentage of the maximal signal observed in WT membrane using 100 µm oxo-M. Data are mean ± SD from *n* = 2 independent experiments.

To study the role of M1Rs in the hippocampus, we used 2 recently described M1R agonists 77-LH-28-1 (Langmead et al. 2008) and GSK compound 5 (GSK-5, [Budzik et al. 2010]). We sought to confirm the selectivity, potency, and efficacy of these 2 compounds on muscarinic receptors. Using recombinant cells expressing the 5 muscarinic receptor subtypes, we found that GSK-5 activated human M1Rs with potency (EC_50_) and efficacy (*E*_max_) values of 19.6 nm and 89 ± 3%, respectively, and displayed some weak agonist activity at M2 and M4 receptors at higher concentrations (EC_50_ and *E*_max_ values of 6346 nm and 39 ± 2% and 8702 nm and 24 ± 8%, respectively; Fig. 1*A*). No agonism of M3 or M5 receptors was observed over the concentration range tested. 77-LH-28-1 activated human M1Rs with EC_50_ and *E*_max_ values of 22.2 nm and 98 ± 2%, respectively (Fig. 1*B*). No agonism of M2–M5 receptors was observed over the concentration range tested. To test antagonist properties of GSK-5 and 77-LH-28-1, the broad-spectrum agonist, acetylcholine, was applied after preincubation with GSK-5 or 77-LH-28-1. In line with its agonist activities, GSK-5 also displayed antagonism at M2 and M4 receptors with IC_50_ 5733 and 3621 nm, respectively (Fig. 1*C*). 77-LH-28-1 displayed antagonism at M2, M4, and M5 receptors with IC_50_ 1188, 2025, and 2220 nm, respectively (Fig. 1*D*). Therefore, GSK-5 and 77-LH-28-1 both are efficacious and potent M1R agonists that display >50-fold selectivity for M1R over the other muscarinic receptor subtypes.

To confirm the potency and efficacy profiles of GSK-5 and 77-LH-28-1 at native rodent M1Rs, GTPɣ[^35^S] binding studies were performed using hippocampal membrane preparations from WT and M1R KO mice (Fig. 1*E*). Concentration response curves for the broad-spectrum muscarinic receptor agonist oxotremorine M (oxo-m) for Gα_q_ activation in WT hippocampus yielded EC_50_ = 171 nm and *E*_max_ = 99 ± 5% in line with previous findings (Watt et al. 2011). No response was observed in tissue from M1R KO mice demonstrating that M1Rs mediate all muscarinic Gq-related activity in hippocampus in this assay. Similarly, concentration response curves for GSK-5- and 77-LH-28-1-mediated Gα_q_ activation in WT hippocampus displayed EC_50_ values of 90 and 156 nm, respectively, and *E*_max_ values of 79 ± 4% and 92 ± 2%, respectively, with no response in M1R KO mice. These results confirm 77-LH-28-1 and GSK-5 as selective M1R agonists and are active at mouse native M1Rs.

### M1R Activation Depolarises Hippocampal CA1 Pyramidal Neurons and Increases E–S Coupling

**Figure 2.**
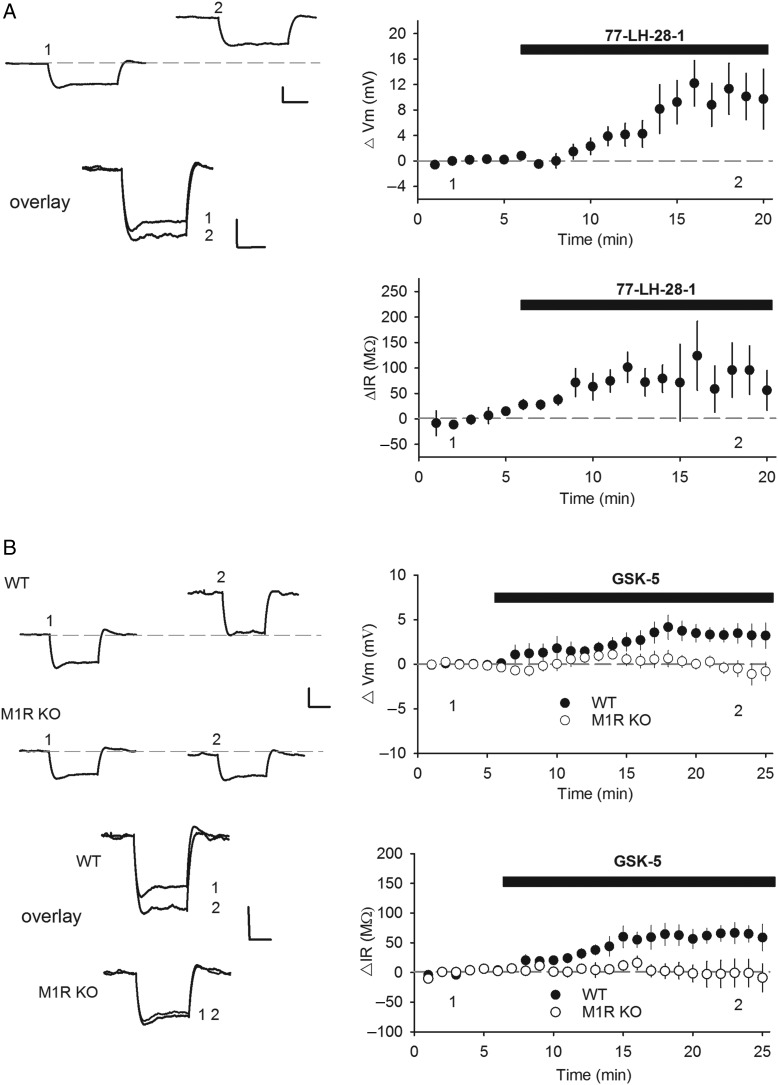
M1R activation enhances CA1 pyramidal cell excitability. (*A*) The M1R agonist 77-LH-28-1 (7 µm) depolarized CA1 pyramidal cells (top) and increased input resistance (bottom) in whole-cell current clamp recordings. (*B*) The M1R agonist GSK-5 (500 nm) depolarized CA1 pyramidal cells (top) and increased input resistance (bottom) in WT but not M1R KO mice. Data are plotted as mean ± s.e.m. Example traces taken from Points 1 and 2 show response to hyperpolarizing current injection (20 pA, 500 ms). Dashed line shows baseline membrane potential. Traces overlaid below for comparison. Scale bars: 2 mV and 200 ms.

There is good evidence that M1R activation underlies the muscarinic receptor-dependent depolarization and increase in input resistance in hippocampal pyramidal neurons (Buchanan et al. 2010; Dasari and Gulledge 2011). We tested this by studying the effects of 77-LH-28-1 or GSK-5 in slices from 6- to 9-week-old mice. CA1 pyramidal cell resting membrane potential and input resistance for our recordings were −65.2 ± 0.8 mV and 71 ± 7 MΩ, respectively, and were consistent across data sets. 77-LH-28-1 (7 µm) depolarized CA1 pyramidal neurons by 10.4 ± 3.9 mV (Fig. 2*A*; *n* = 8, *P* = 0.015) and increased input resistance by 86 ± 51 MΩ (Fig. 2*B*; *n* = 8, *P* = 0.044), consistent with previous data in slices from 2-week-old rats (Buchanan et al. 2010). Similarly, application of GSK-5 (500 nm) depolarized CA1 pyramidal cells by 3.3 ± 1.1 mV (Fig. 2*C*; *n* = 8, *P* = 0.030) and increased input resistance by 63 ± 16 MΩ (Fig. 2*D*; *n* = 8, *P* = 0.0095). The increase in input resistance was indistinguishable, but 77-LH-28-1 caused a greater depolarization than GSK-5 (*P* = 0.039). Importantly, GSK-5 had no effect on these parameters in interleaved slice experiments from M1R KO mice performed blind to genotype (Fig 2*C*,*D*; −0.5 ± 0.9 mV, −3 ± 23 MΩ, *n* = 7). These findings confirm that selective activation of M1Rs causes an increase in input resistance and a depolarization of CA1 pyramidal neurons.

**Figure 3.**
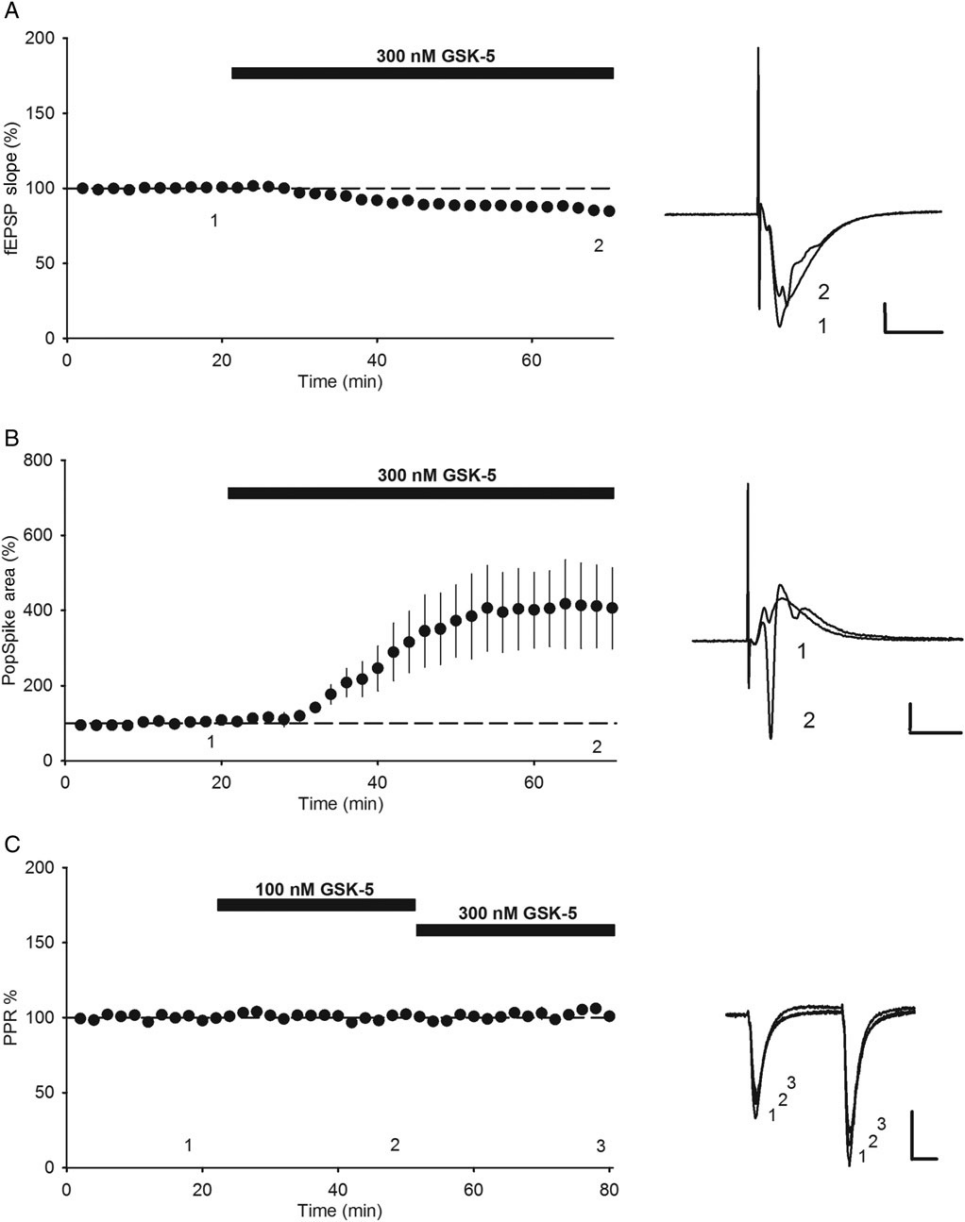
M1R activation increases EPSP-spike coupling. (*A*) GSK-5 (300 nm) caused a small depression in fEPSP slope in extracellular field potential recordings in stratum radiatum. (*B*) GSK-5 (300 nm) increased population spike area recorded in stratum pyramidale. (*C*) 100 nm or 300 nm GSK-5 did not alter paired-pulse ratio. Data are plotted as mean ± s.e.m. Example field potential traces taken from Points 1, 2, and 3 as indicated. Scale bars: 0.5 mV and 10 ms.

An M1R-dependent increase in input resistance and depolarization is predicted to increase cellular excitability and thus increase the spike output of CA1 pyramidal neurons in response to excitatory synaptic input, also termed EPSP-spike (E–S) coupling. To test this idea, extracellular field potential recordings were made simultaneously from stratum radiatum and stratum pyramidale in the CA1 region of hippocampal slices from 6- to 9-week-old rats to record the synaptic input and spike output, respectively, in the same experiment. GSK-5 (300 nm) caused a small depression of the fEPSP slope recorded in stratum radiatum (Fig. 3*A*; 86.5 ± 1.0%, *n* = 6, *P* = 0.0014), but a large increase in the population spike area recorded in stratum pyramidale (Fig. 3*B*; 391.9 ± 89.9%, *n* = 6, *P* = 0.0033). These M1R-mediated effects are unlikely to be due to changes in presynaptic function since paired-pulse ratio was unaltered following application of GSK-5 (Fig. 3*C*; *n* = 4, *P* = 0.14, average paired-pulse ratio 1.66 ± 0.04). These data therefore show that the increase in CA1 pyramidal cell excitability caused by M1R activation results in a robust increase in spike output of CA1 pyramidal neurons.

### M1R Activation Potentiates CA1 Synaptic Transmission in an NMDAR-Dependent Mechanism That Bi-directionally Occludes LTP

**Figure 4.**
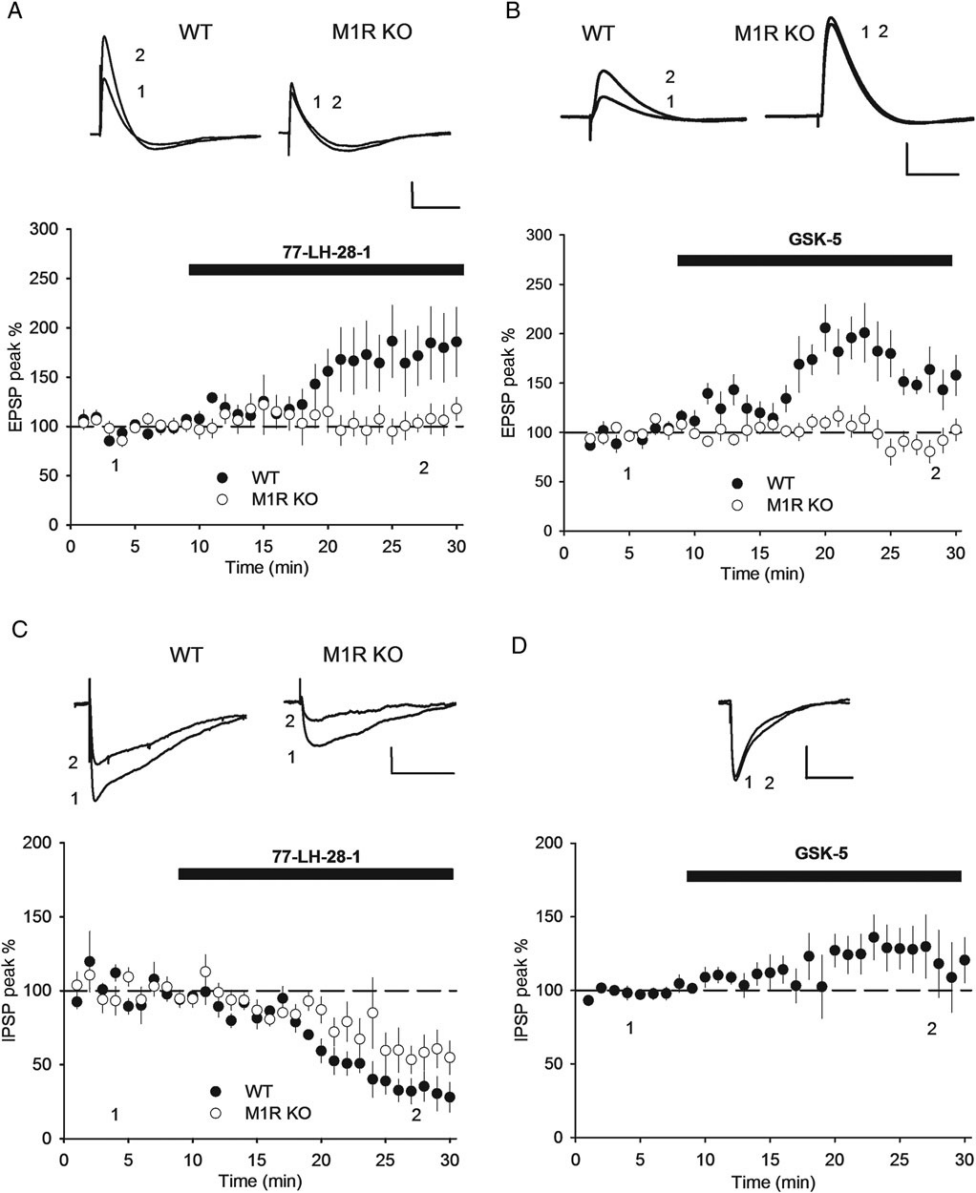
M1R activation increases EPSP, but not IPSP amplitude. (*A*) 77-LH-28-1 (7 µm) increased EPSP peak in WT but not M1R KO mice in whole-cell current clamp recordings in the presence of GABA_A_ and GABA_B_ receptor antagonists. (*B*) GSK-5 (500 nm) increased EPSP peak in WT but not M1R KO mice. (*C*) 77-LH-28-1 (7 µm) decreased IPSP peak in WT and M1R KO mice recorded in the presence of AMPA and NMDAR antagonists. (*D*) GSK-5 (500 nm) did not change IPSP peak in WT mice. Data plotted as mean ± s.e.m. Example voltage traces in response to synaptic stimulation taken from Points 1 and 2 as indicated. Scale bars: 2 mV (*A–C*) or 4 mV (*D*) and 100 ms (*A,B*) or 200 ms (*C,D*).

We next further investigated the role of M1Rs in regulating synaptic transmission using whole-cell patch-clamp recordings from CA1 pyramidal neurons from 6- to 9-week-old mice. Both 77-LH-28-1 (7 µm) and GSK-5 (500 nm) increased the amplitude of pharmacologically isolated EPSPs in CA1 pyramidal neurons (Fig. 4*A*,*B*; GSK-5, *P* = 0.00025; 77-LH-28-1, *P* = 0.028). This was due to M1R activation since no increase was seen in slices from M1R KO mice (77-LH-28-1: WT 194.2 ± 42.0%, *n* = 6; M1R KO 118.5 ± 24.7%, *n* = 6, *P* < 0.028; GSK-5: WT 170.4 ± 21.2%, *n* = 7; M1R KO 95.0 ± 11.9%, *n* = 6, *P* < 0.00057). In these experiments, membrane potential was maintained constant with current injection of ∼50–100 pA during the application of M1R agonist, which limits the increase in input resistance (Buchanan et al. 2010; Petrovic et al. 2012). Therefore, it is unlikely that the increase in EPSP amplitude could be caused by an increase in input resistance. We also tested the effects of M1R activation on inhibitory synaptic transmission onto CA1 pyramidal neurons. 77-LH-28-1 (7 µm) depressed pharmacologically isolated IPSPs in slices from WT mice (Fig. 4*C*; 39.1 ± 9.2%, *n* = 6, *P* = 0.0013) whereas GSK-5 did not have any effect (Fig. 4*D*; 124.4 ± 17.2%, *n* = 7, *P* = 0.57). Experiments on slices from M1R KO mice showed that 77-LH-28-1 still decreased IPSP amplitude in the absence of M1Rs (Fig. 4*C*; 64.0 ± 13.3%, *n* = 6, *P* = 0.018) demonstrating that this effect on inhibitory transmission was due to an off-target action of 77-LH-28-1. Taken together, these data show that M1R activation increases the strength of excitatory input onto CA1 pyramidal neurons but has no effect on inhibitory transmission. It is important to note that the lack of potentiation of fEPSPs by M1R agonist in the extracellular recordings is not inconsistent with the observed EPSP potentiation in the patch-clamp recordings. The lack of ability to control membrane potential during extracellular recordings, combined with the large increase in excitability caused by M1R activation, suppresses the ability to detect the increase in EPSP amplitude in extracellular recordings.

**Figure 5.**
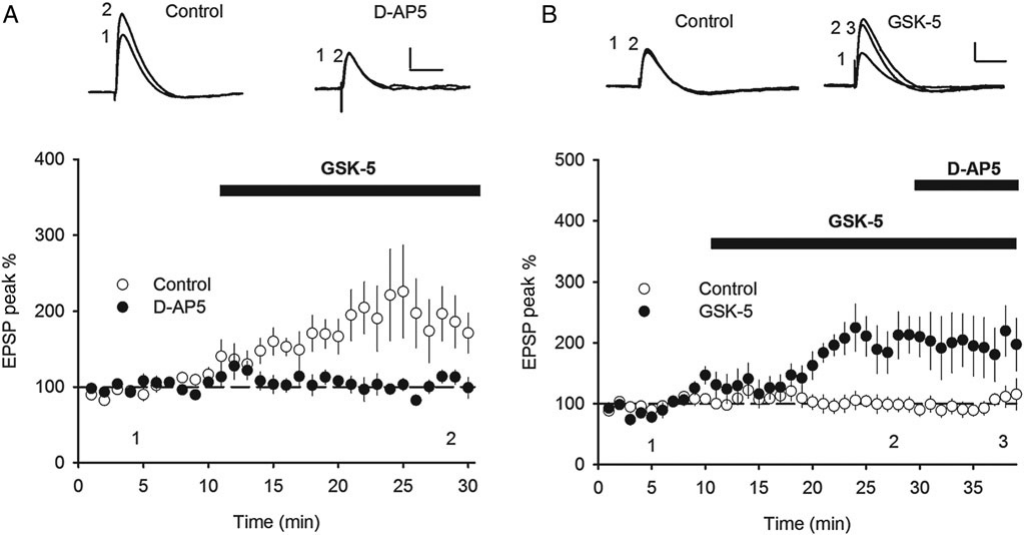
M1R-induced EPSP enhancement is NMDAR dependent. (*A*) The NMDAR antagonist D-AP5 (50 µm) prevented the increase in EPSP amplitude induced by application of GSK-5 (500 nm) in whole-cell current clamp recordings. (*B*) Application of D-AP5 (50 µm) after GSK-5 (500 nm) failed to reverse the increase in EPSP amplitude induced by GSK-5. Data plotted as mean ± s.e.m. Example voltage traces in response to synaptic stimulation taken from Points 1 or 2 as indicated. Scale bars: 2 mV and 50 ms (*A,B*).

Activation of muscarinic receptors facilitates LTP or, at high agonist concentrations, can induce long-term depression (Boddeke et al. 1992; Ovsepian et al. 2004; Shinoe et al. 2005; Scheiderer et al. 2006; Seol et al. 2007; Dickinson et al. 2009; Buchanan et al. 2010; Gu and Yakel 2011; Connor et al. 2012; Digby et al. 2012; Navakkode and Korte 2012), likely through the activity at a number of muscarinic receptor subtypes (Teles-Grilo Ruivo and Mellor 2013). The selective activation of M1Rs is shown to enhance NMDAR activity, which facilitates the induction of LTP (Marino et al. 1998; Sur et al. 2003; Shinoe et al. 2005; Buchanan et al. 2010). Therefore, we hypothesized that the long-lasting M1R-induced increase in EPSP amplitude may share certain mechanisms with LTP. We first tested whether the M1R-induced increase in EPSP amplitude was dependent on NMDAR activation. Recordings of excitatory synaptic transmission were made from CA1 pyramidal cells held at −70 mV in current clamp in the presence or absence of the NMDAR antagonist D-AP5 (50 µm). In the absence of D-AP5, GSK-5 (500 nm) caused an increase in EPSP amplitude; however, in interleaved experiments, co-application of D-AP5 starting at least 10 min before GSK-5 prevented the potentiation (Fig. 5*A*; GSK-5 alone: 196.3 ± 41.6%, *n* = 10; GSK-5 + D-AP5: 101.2 ± 10.3%, *n* = 9, *P* = 0.030). Similar to 77-LH-28-1 (Buchanan et al. 2010), GSK-5 also prolonged the duration of EPSPs (EPSP half-width 141.2 ± 25.3%, *P* = 0.025). We next tested whether the potentiation was due to an increase in the NMDAR-mediated component of the EPSP. However, the GSK-5-induced increase in EPSP amplitude was not reversed when D-AP5 was applied after the GSK-5-induced potentiation had been established (Fig. 5*B*). These results demonstrate that M1R activation triggers an NMDAR-dependent increase in EPSP amplitude that is expressed as an increase in the AMPAR-mediated EPSP.

**Figure 6.**
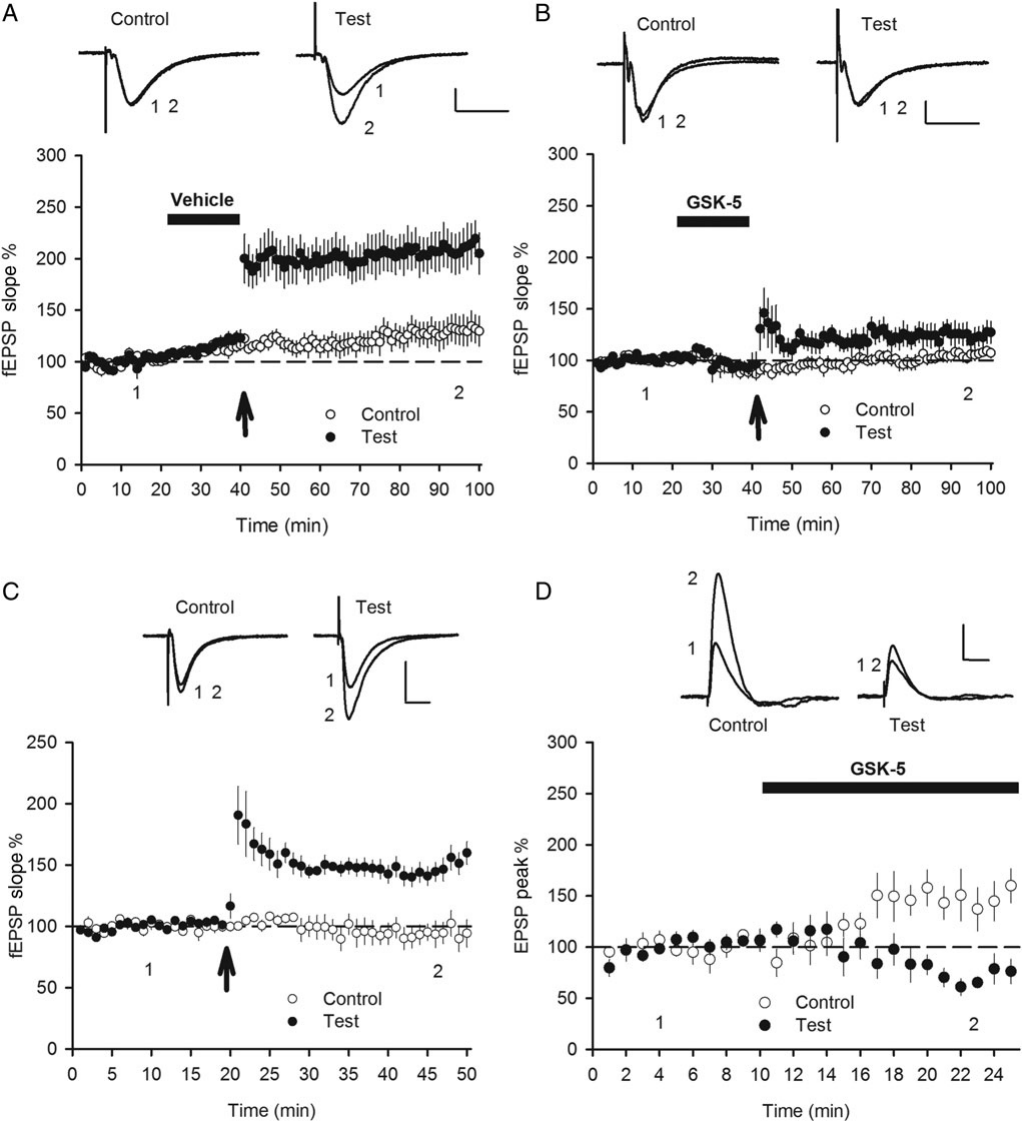
M1R-induced EPSP enhancement bi-directionally occludes with LTP. (*A*) Theta burst stimulation (arrow) induced pathway-specific LTP after a 20-min application of vehicle (DMSO) in extracellular field potential recordings from stratum radiatum. (*B*) Theta burst stimulation (arrow) failed to induce LTP after a 20-min application of GSK-5 (500 nm). (*C*) Extracellular recording demonstrated pathway-specific LTP induction by theta burst stimulation (arrow) in the test pathway. (*D*) In the same slices as (*C*), subsequent whole-cell recording from CA1 pyramidal cells showed that GSK-5 (500 nm) caused an increase in EPSP amplitude only in the control synaptic pathway that did not receive theta burst stimulation. Data plotted as mean ± s.e.m. Example field potential and voltage traces in response to synaptic stimulation taken from Points 1 or 2 as indicated. Scale bars: 0.2 mV and 10 ms (*A*, *B*), 0.5 mV and 10 ms (*C*), 2 mV and 50 ms (*D*).

Since M1R activation produces an NMDAR-dependent increase in synaptic transmission, we next tested whether the potentiation occludes LTP. To measure Schaffer collateral LTP, we made extracellular field potential recordings in the stratum radiatum and stimulated 2 independent synaptic pathways with LTP being induced in the test pathway using a theta burst protocol. In interleaved experiments, vehicle (DMSO) or GSK-5 (500 nm) was applied to the slice for 20 min prior to LTP induction. After application of vehicle, theta burst stimulation induced robust LTP in the test pathway (Fig. 6*A*; test pathway 206.9 ± 18.9% vs. control pathway 128.4 ± 12.7%, *n* = 8, *P* = 0.00035). However, after application of 500 nm GSK-5, theta burst stimulation failed to induce LTP in the test pathway (Fig. 6*B*; test pathway 124.1 ± 10.4%, *n* = 8 vs. control pathway 103.5 ± 5.3%, *n* = 8, *P* = 0.074). Similarly, application of 100 nm GSK-5 also prevented the induction of LTP by theta burst stimulation (data not shown; test pathway 101.0 ± 4.6%, *n* = 3 vs. control pathway 110.8 ± 7.5%, *n* = 3, *P* = 0.59). Therefore, prior activation of M1Rs prevents subsequent induction of LTP. Finally, we tested whether prior induction of LTP occludes with the M1R-induced potentiation. In two-pathway experiments, we first induced LTP using extracellular recording and then subsequently monitored effects of GSK-5 on the same synaptic pathways in individual CA1 pyramidal neurons using whole-cell patch-clamp recording. Theta burst stimulation in the test pathway induced a pathway-specific LTP (Fig. 6*C*; test pathway 146.7 ± 7.4% vs. control pathway 95.1 ± 9.4%, *n* = 8, *P* = 0.00059). GABA_A_ and GABA_B_ receptor antagonists picrotoxin (50 µm) and CGP55845 (1 µm) were then applied and a whole-cell recording made from a CA1 pyramidal cells in current clamp in the same region of the slice within 40 min of the application of theta burst stimulation. The effect of GSK-5 addition on synaptic transmission on the 2 pathways was then monitored. Application of GSK-5 (500 nm) caused an increase in EPSP amplitude in the control pathway (that had not undergone LTP induction), but importantly no potentiation was observed in the test pathway at which LTP had been induced (Fig. 6*D*; GSK-5 potentiation: control pathway 142.2 ± 18.9% vs. test pathway 70.8 ± 10.4%, *n* = 8, *P* = 0.0012). Taken together, these findings show that the M1R-dependent potentiation of synaptic transmission bi-directionally occludes with LTP.

## Discussion

In this study, we demonstrate that activation of M1Rs in adult hippocampus causes a long-lasting enhancement of excitatory synaptic transmission that bears the hallmarks of LTP: It is expressed as an increase in AMPAR-mediated transmission, it is NMDAR dependent, and it bi-directionally occludes with LTP. This novel mechanism for synaptic potentiation occurs in addition to the more well-described action of M1Rs to increase the excitability of CA1 pyramidal neurons, which we also confirm in the present study.

One important issue that has dogged our understanding of the specific roles of the different muscarinic receptors in the CNS has been the lack of well-characterized subtype selective ligands. In the present study, we confirm that GSK-5 and 77-LH-28-1 are potent M1R agonists with sufficient selectivity over the other muscarinic receptor subtypes to be useful tools both in vitro and potentially in vivo. The availability of muscarinic receptor KO mice has additionally been very important in the present study to confirm the on-target actions of these molecules. Thus, we definitively ascribe the following actions in adult hippocampal CA1 pyramidal neurons to M1R agonism: depolarization, increase in input resistance, increase in E–S coupling, potentiation of AMPAR-mediated synaptic transmission requiring NMDAR activity. We note that one observed action of 77-LH-28-1, that of depressing inhibitory synaptic transmission, is not related to its activity at M1Rs, and we do not know the mechanism for this effect.

The orthosteric binding site is highly conserved across muscarinic receptors (Haga et al. 2012); hence, the search for subtype selective agonists has recently shifted toward agonists binding to allosteric sites, of which AC-42 was the first characterized for the M1 receptor (Spalding et al. 2002). The 2 M1R-selective allosteric agonists used in this study, GSK-5 and 77-LH-28-1, are derivatives of AC-42, have similar structures, and are presumed to bind to the same extracellular region of the receptor although the mechanism of binding is yet to be defined (Langmead et al. 2008; Budzik et al. 2010). GSK-5 and 77-LH-28-1 both exhibit >10^2^-fold selectivity for M1Rs over other muscarinic receptor subtypes (Fig. 1), and their effects on cellular excitability and synaptic transmission are almost entirely absent in M1R KO mice (Figs 2 and 4 and [Buchanan et al. 2010]). The only exception is the depression of IPSPs by 77-LH-28-1. It is not clear what this off-target action is but activation of muscarinic M2 receptors is known to depress inhibitory synaptic transmission (Szabo et al. 2010) so it is possible that 77-LH-28-1 has some activity at M2 receptors although this is not suggested by the data in Figure 1. In addition, we note that GSK-5 is reported to have a good overall general selectivity profile as assessed in the CEREP panel (Budzik et al. 2010) and 77-LH-28-1 is reported to possess some cross-reactivity at dopamine D2 and 5-HT_2B_ receptors (Melancon et al. 2013).

The effects of M1R agonism on CA1 pyramidal cell excitability we observe are consistent with several previous studies (Langmead et al. 2008; Buchanan et al. 2010; Dasari and Gulledge 2011) and are confirmed by showing the effects are lost in slices from M1R KO mice. M1Rs cause a depolarization by inhibiting Kv7 potassium channels, which also produces an increase in input resistance (Dutar and Nicoll 1988). We now show that this has the effect of dramatically increasing E–S coupling and hence spike output of CA1 pyramidal neurons. Furthermore, it is likely that the potentiation of excitatory synaptic transmission in adult slices that we observe also contributes to this increase in spike output observed in our extracellular recordings.

The potentiation of AMPAR-mediated EPSPs by M1Rs that we describe is a novel and interesting phenomenon. A number of previous studies have shown that muscarinic agonism, for example using carbachol, causes a depression in excitatory synaptic transmission onto CA1 pyramidal neurons, including work from some of the present authors (Isaac et al. 2009). However, our results and those from recent studies strongly indicate that the depression in transmission is independent of M1 and is mediated by M4 muscarinic receptors via a presynaptic mechanism (Buchanan et al. 2010; Dasari and Gulledge 2011). The M1R-dependent potentiation we observe appears to be distinct from the short-term M4-dependent regulation of transmission; it is postsynaptic and uses induction and expression mechanisms in common with CA1 LTP.

Previous work shows that M1R activation leads to an increase in NMDAR function by inhibiting SK potassium channels that negatively regulate NMDAR activity (Ngo-Anh et al. 2005; Bloodgood and Sabatini 2007; Buchanan et al. 2010; Giessel and Sabatini 2010). The SK channel inhibition by M1Rs removes this negative regulation promoting NMDAR activity. This model is supported by the requirement for NMDAR activation in studies where M1Rs facilitate LTP induction (Anagnostaras et al. 2003; Shinoe et al. 2005; Buchanan et al. 2010) and fits more broadly with the actions of muscarinic receptors in facilitating the induction of spike timing-dependent LTP (Adams et al. 2004; Sugisaki et al. 2011). We now show that M1R agonist application to slices causes a potentiation of AMPAR-mediated EPSPs that depends on NMDAR activity. Thus, our findings suggest that the M1R-dependent increase in NMDAR activation due to suppression of SK causes a potentiation of AMPAR-mediated synaptic transmission onto CA1 pyramidal neurons. Further, this process shares its mechanism of expression with LTP because it bi-directionally occludes with LTP. It is possible that the M1R-dependent potentiation also shares other mechanisms with LTP; future work will be needed to explore this possibility.

Activation of M1Rs has also been shown to cause a potentiation of glutamatergic synaptic transmission onto CA1 pyramidal neurons via activation of IP3 receptors leading to insertion of AMPARs into spines (Markram and Segal 1992; Fernandez de Sevilla et al. 2008; Fernandez de Sevilla and Buno 2010). This model is supported by the observed muscarinic receptor-mediated increase in dendritic Ca^2+^ and subsequent activation of CAMKII (Muller and Connor 1991; Muller et al. 1992). Although superficially similar to the potentiation we describe, the potentiation described by Fernandez de Sevilla and coworkers has 2 important differences: Its induction does not require NMDARs and it does not occlude with LTP. Moreover, it is observed in slices from 2-week-old rats, an age which we find no potentiation of synaptic transmission by M1R agonist (Buchanan et al. 2010). Thus, at present, the relationship between these 2 forms of muscarinic receptor-dependent plasticity is unclear.

In experimental systems where muscarinic receptor activation is required for induction of spike timing-dependent LTP, it is also shown that prolonged exposure to muscarinic receptor agonists may desensitize receptors thereby preventing subsequent facilitation of LTP (Muller et al. 1988; Adams et al. 2004). This mechanism is unlikely to explain the lack of LTP found after application of GSK-5 (Fig. 6) since the increase in synaptic strength produced by GSK-5 or theta burst stimulation bi-directionally occludes with one another.

In vivo, the induction of synaptic plasticity in the hippocampus occurs within the context of ongoing network activity, which is regulated by acetylcholine release. Rhythmic network activity at theta frequency is strongly associated with elevated acetylcholine release in both awake behavior and REM sleep (Lee et al. 1994; Marrosu et al. 1995; Zhang et al. 2010). Gamma frequency oscillations are often nested within theta activity and can be elicited in slice preparations by application of muscarinic agonists (Bragin et al. 1995; Fisahn et al. 1998). Conversely, hippocampal sharp wave ripple activity that occurs during non-REM sleep and quiet wakefulness is depressed by acetylcholine release (Vandecasteele et al. 2014). All 3 of these network oscillations have been strongly implicated in coordinating neuronal firing within the timeframes required for the induction of synaptic plasticity (Huerta and Lisman 1993; Kwag and Paulsen 2009; Sadowski et al. 2011). Our data do not directly address the role of network oscillations on synaptic plasticity or the impact of M1R activation on this interaction since our slices did not exhibit oscillatory activity even in the presence of M1R agonists. However, the potentiation of excitatory synaptic transmission by M1Rs will provide another mechanism by which acetylcholine regulates hippocampal network dynamics.

Acetylcholinesterase inhibitors are the only major treatment available for the cognitive deficits associated with Alzheimer's disease, but there are significant side effects associated with their use. The development of muscarinic receptor subtype selective agonists or potentiators aims to provide better treatment for the cognitive deficits in diseases such as Alzheimer's and schizophrenia, while limiting the side effects (Hasselmo and Sarter 2011). A clinical study using GSK1034702, a close analogue of GSK-5, has recently reported positive effects on cognition (Nathan et al. 2013). Our study now provides one potential mechanism for this pro-cognitive effect through lowering the threshold for induction of hippocampal LTP and an increase in spike output of pyramidal neurons. Indeed, an in vivo electrophysiological study shows that GSK5 produces increased pyramidal cell firing in rat hippocampus in anesthetized rats (Budzik et al. 2010). In studies not reported here, we have also found that either GSK5 or GSK1034702 causes an increase in CA1 pyramidal cell firing in vivo and that this is reversed by the broad-spectrum muscarinic receptor antagonist, scopolamine. However, the relationship of the M1R-induced synaptic enhancement and increase in spike output to hippocampal network function and cognition are likely complex. Across a circadian cycle, the hippocampal states and therefore neuromodulatory input necessary for optimal memory encoding vary such that M1R agonism may prove more beneficial under certain behavioral states. This complex issue requires considerable further work to address and will be of great interest to understand.

## Funding

S.H.D. was funded through the Lilly Centre for Cognitive Neuroscience. J.R.M. was funded through the Wellcome Trust and Biotechnology and Biological Sciences Research Council, U.K. Funding to pay the Open Access publication charges for this article was provided by the Biotechnology and Biosciences Research Council (BBSRC) and the Wellcome Trust.
